# Synthesis and Evaluation of the Anti-Microbial Activity of New Heterocycles Containing the 1,3,4-Thiadiazole Moiety

**DOI:** 10.3390/molecules161210420

**Published:** 2011-12-15

**Authors:** Thoraya A. Farghaly, Magda A. Abdallah, Zienab A. Muhammad

**Affiliations:** Department of Chemistry, Faculty of Science, Cairo University, Giza 12613, Egypt

**Keywords:** antimicrobial activity, enaminone, 1,3,4-thiadiazole, nitrogen nucleophiles, carbon nucleophiles

## Abstract

A new series of thiadiazole-enaminones **4** were synthesized via reactions of 5-acetyl-1,3,4-thiadiazoles **3** with dimethylformamide-dimethylacetal (DMF-DMA). The simple phenyl substituted thiadiazole-enaminone **4f** was used as a synthetic precursor for the preparation of a wide variety of new heterocyclic compounds, including the 5-substituted-1,3,4-thiadiazole derivatives **5**, **6**, **11**, **12** and **13**, which were obtained via reactions of **4f** with nitrogen nucleophiles. Also, reactions of enaminone **4f** with carbon nucleophiles afforded the respective 1,3,4-thiadiazoles **8a–d**. In addition, the results of the antimicrobial activities of thiadiazole-enaminones **4** and their precursors **2** and **3 **indicate that some members of this series display promising activities against all tested microorganisms.

## 1. Introduction

Substituted 1,3,4-thiadiazoles have attracted considerable interest owing to their wide spectrum biological activity, including antimicrobial, antituberculosis, anesthetic, antithrombotic, anticonvulsant, antihypertensive, anti-inflammatory and antiulcer properties [1,2,3,4,5]. Enaminones are polydentate reagents that have been utilized extensively in this decade as building blocks in organic synthesis [6,7,8,9,10,11,12]. In continuation of our previous reports on synthesis of bioactive heterocyclic compounds [10,11,12,13,14,15,16] in this investigation we have prepared a new series of enaminone-linked 1,3,4-thiadiazoles and investigated their chemical reactivity with a variety of nucleophilic reagents. In addition, we tested the biological activity of the resulting thiadiazole derivatives against select microorganisms.

## 2. Results and Discussion

**Scheme 1:**
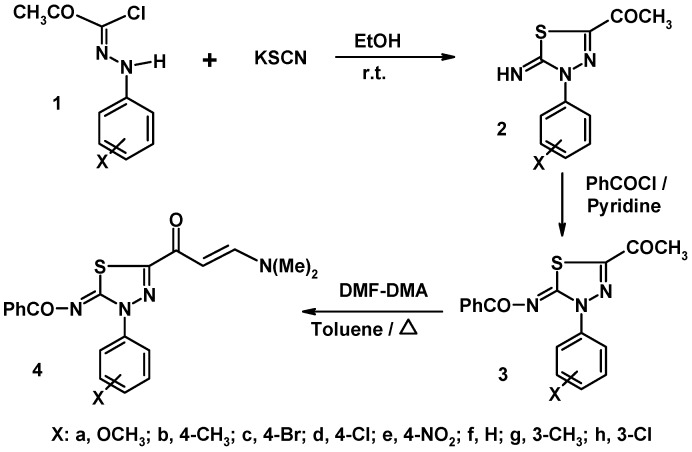
Synthesis of enaminones **4a–h**.

The new enaminone linked 1,3,4-thiadiazoles **4a–h** were prepared by reaction of the corresponding 5-acetyl-2-benzoylimino-3-aryl-1,3,4-thiadiazoles **3a–h** with dimethylformamide-dimethylacetal (DMF-DMA) under reflux in dry toluene (Scheme 1). The structures of the products were established based on their elemental and spectral data. For example, the ^1^H-NMR spectra of these products contained two singlet signals at *δ* 5.8 and 8.1 ppm (*J* = 12 Hz) which correspond to the two trans-olefinic protons in the *E*-enaminone moieties [16,17].

**Scheme 2:**
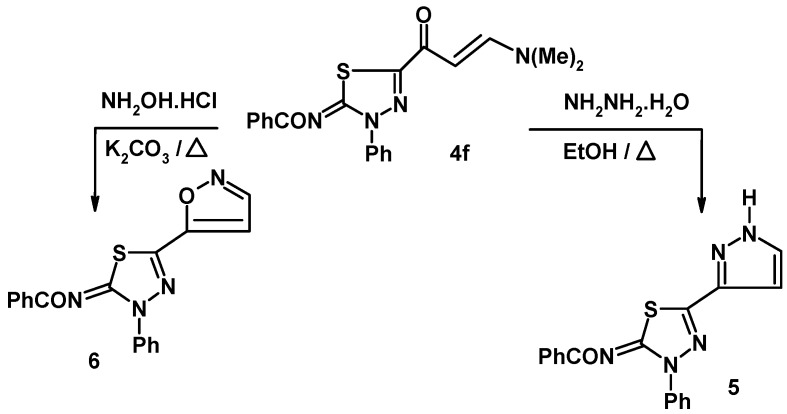
Reactions of enaminone **4f** with hydrazine hydrate and hydroxylamine.

**Scheme 3:**
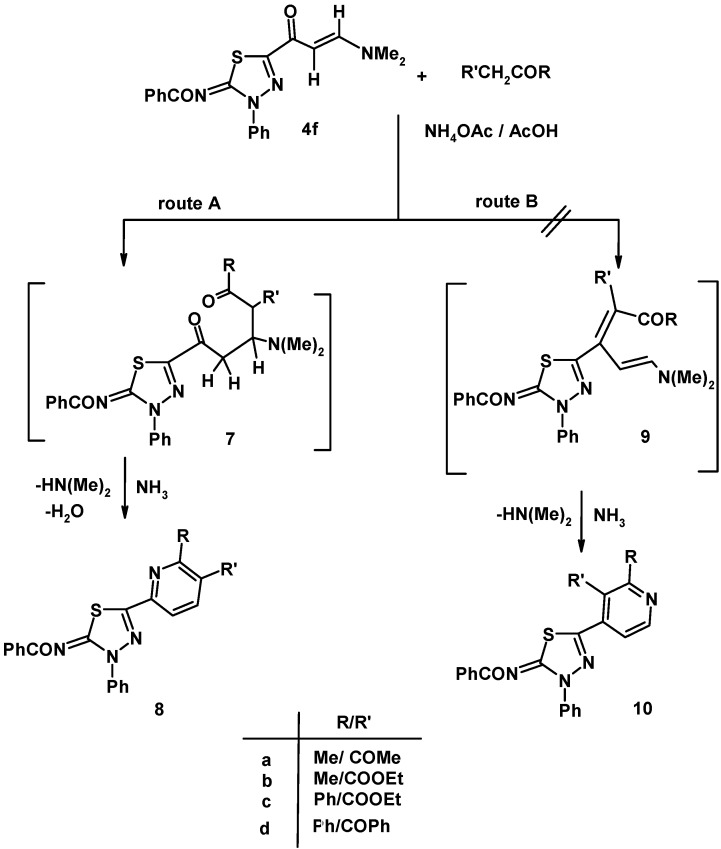
Reaction of enaminone **4f** with active methylene compounds.

Reaction of enaminone **4f** with hydrazine hydrate in ethanol under reflux led to formation of the thiadiazole-pyrazole linked product **5** (Scheme 2) whose structure was assigned using spectroscopic and elemental analysis methods. For example, the IR spectrum of this compound contains a carbonyl band at 1,607 cm^−1^ attributed to the benzamide group and it does not contain an enaminone group-associated carbonyl band. Also, no olefinic or methyl proton resonances were observed in the ^1^H-NMR spectrum of **5**, which did contain a singlet at *δ* 9.21 ppm due to the pyrazolyl-NH proton. In a related manner, reaction of enaminone **4f** with hydroxylamine hydrochloride in ethanol in the presence of potassium carbonate led to formation of the thiadiazole-isoxazole **6**. The structure of the latter compounds was also established based on both elemental and spectral data (see Experimental). The reactivity of enaminone **4f** towards several C- nucleophiles was explored next. Compound **4f** reacts with active methylene compounds in acetic acid in the presence of ammonium acetate under reflux to afford products that could have either of the regioisomeric linked thiadiazole-pyridine structures represented by either **8** or **10**. Two pathways are outlined in Scheme 3 for this reaction. The reaction may proceed by initial Michael addition (route A) of the active methylene compound to the activated double bond of **4f** to give the Michael adduct **7** followed by tandem elimination of dimethylamine and condensation with ammonia to give product **8** or the other suggested pathway (route B) may proceed by initial condensation of active methylene compound with the carbonyl group of **4f** which leads to formation of intermediate **9** that cyclizes in the presence of ammonium acetate to give **10**. The latter product **10** was discarded however based on its ^1^H-NMR spectral data. For example, the ^1^H-NMR spectrum of compound **8a** revealed two singlet signals at *δ* 2.49 and 2.63 ppm assigned to the methyl and acetyl protons, in addition to two doublets at *δ* 8.04 and 8.39 ppm (*J* = 7–8 Hz) assigned to pyridine H-3 and H-4. Such value of coupling constant *J* is characteristic for pyridine H-3 and H-4 and much higher than that for H-2 and H-3 (*J* = 4–6 Hz) [18,19] in structure **10** (Scheme 3).

**Scheme 4:**
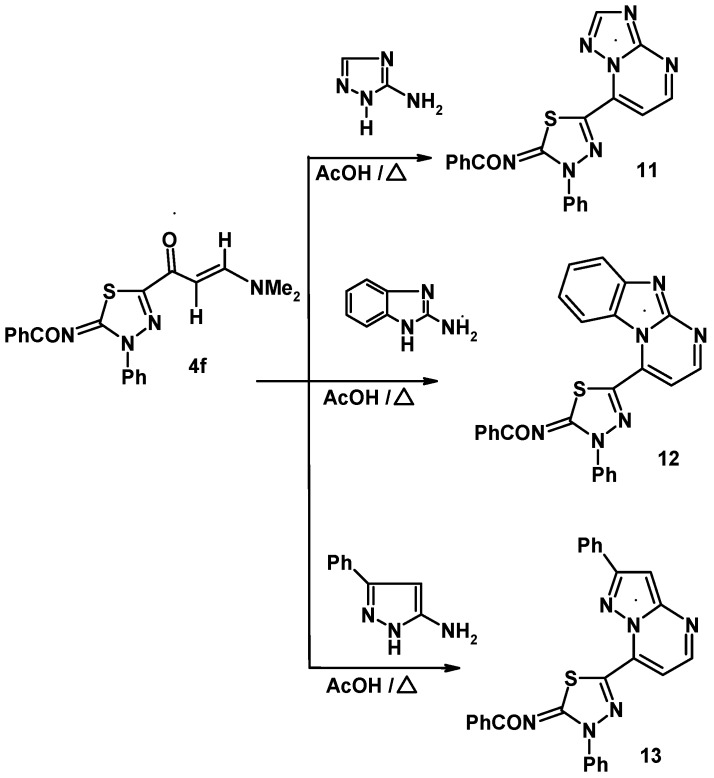
Reaction of enaminone **4f** with heterocyclic amines.

Also, the reactivity of the enaminone **4f** towards some heterocyclic amines was examined. For example, 5-amino-1,2,4-triazole was found to react with **4f** in acetic acid to yield the 1,2,4-triazolo[1,5-a]pyrimidine derivative **11** (Scheme 4). Similarly, treatment of **4f** with each of 2-aminobenzimidazole and 5-amino-3-phenylpyrazole under the same reaction conditions afforded the respective benzimidazo[1,2-a]pyrimidine **12** and pyrazolo[1,5-a]pyrimidine derivatives **13** (Scheme 4). The ^1^H-NMR spectrum of each of the products **11**, **12** and **13** contains two doublets in the regions7.93–8.11 ppm and 8.08–9.04 ppm with *J* values of 4.5 Hz that are assignable to the two vicinal protons in the pyrimidine moieties [20,21].

**Scheme 5:**
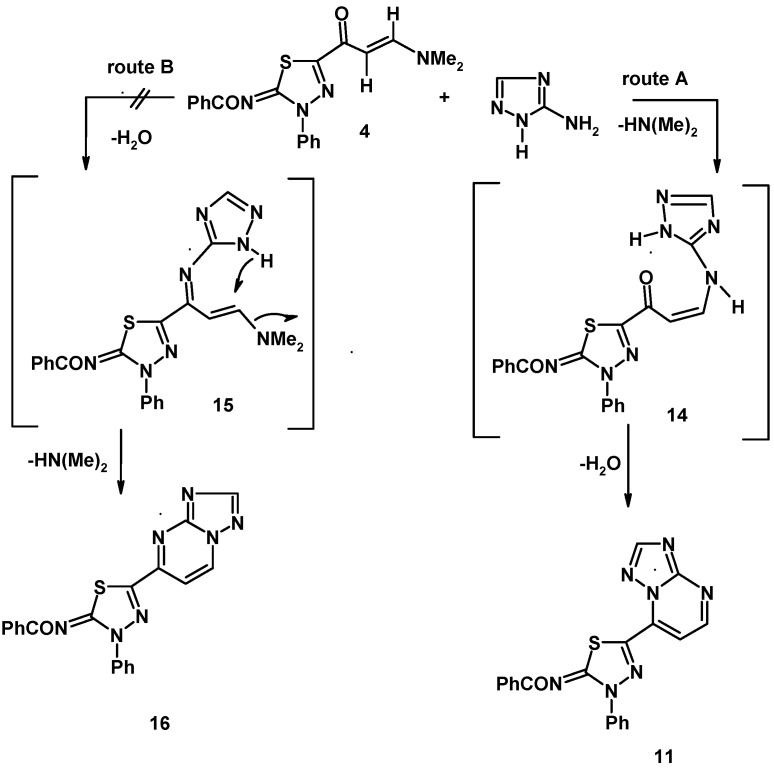
The mechanism of reaction of enaminone **4f** with heterocyclic amine.

A plausible mechanistic pathway for formation of **11**, **12** and **13** involves Michael addition of the exocyclic amino group of the amines to the enaminone double bond of **4f** followed by *in situ* tandem elimination of dimethylamine and dehydrative cyclization (route A) (Scheme 5). Another route (B), producing regioisomer **16** via intermediate **15** does not operate in this process.

### 2.1. Biological Screening Anti-Microbial Activities

*In vitro* anti-microbial screening of the compounds **2**, **3** and **4** prepared in this study was carried out using fourfungal strains, including *Aspergillus fumigatus* RCMB 002003 (AF), *Geotrichum candidum* RB 052006 (GC)*, Candida albicans* RCMB 005002 (CA) and *Syncephalastrum racemosum* RCMB 005003(SR), and four bacterial species, including the Gram positive bacteria *Staphylococcus aureus* RCMB 000106 (SA) and *Bacillus subtilis* RCMB 000107 (BS), and the Gram negative bacteria *Pseudomonas aeruginosa* RCMB 000102 (PA) and *Escherichia coli* RCMB 000103 (EC). The results of the investigations with the thiadiazole derivatives **2a–h** (Table 1 and Table 2) showed that **2b** displays high activities against all the tested microorganisms. This finding suggests that the presence of an electron-donating C-4 methyl group in the phenyl ring linked to the 1,3,4-thiadiazole moiety promotes increased biological activity. In addition, compound **3c** showed high activities against all tested microorganisms, especially AF, when compared to the standard fungicides itraconazole and clotrimazole. In addition, **3c** showed high activity against all tested bacteria species, especially BS, when compared with the standard bactericides penicillin G and streptomycin. The data obtained by probing the antimicrobial activities of enaminones **4** are given in Table 3, Table 4 and Table 5. The results indicate that **4c** is highly potent against all tested microorganisms. Based on these results, we can conclude that the presence of a bromine substituent at the C-4 of the phenyl group linked to the 1,3,4-thiadiazole moiety causes increased antimicrobial activity.

**Table 1:** Anti-microbial activities.

Microorganism	2a	2b	2c	2d	2e	2f	ST (30 µg/mL)	
*Fungi*							Itraconazole	Clotrimazole
*AF*	12.5	22.3	15.9	20.1	15.2	11.5	28.5	26.1
*GC*	12.5	19.7	18.4	18.3	18.4	12.9	27.1	23.1
*CA*	11.0	19.4	15.2	15.4	15.4	10.7	26.1	18.3
*SR*	NA	18.4	NA	8.2	NA	NA	22.3	20.5
*Gram Positive Bacteria*							Penicillin G	Streptomycin
*SA*	13.4	22.04	18.2	20.4	12.4	11.2	29.4	25.1
*BS*	15.3	24.08	19.3	21.1	13.5	12.3	32.5	29.1
*Gram negative Bacteria*							Penicillin G	Streptomycin
*PA*	NA	22.9	NA	16.2	NA	NA	28.3	24.3
*EC*	10.4	21.9	9.4	18.9	8.6	9.8	33.5	25.6

**Table 2:** Anti-microbial activities.

Microorganism	2g	2h	2i	3a	3b	3c	ST (30 µg/mL)	
*Fungi*							Itraconazole	Clotrimazole
*AF*	19.2	19.2	18.3	13.3	11.2	25.3	28.5	26.1
*GC*	20.4	20.4	18.2	12.4	10.4	24.4	27.1	23.1
*CA*	17.4	18.2	17.1	11.2	9.4	17.2	26.1	18.3
*SR*	16.4	14.4	12.0	9.4	NA	19.9	22.3	20.5
*Gram Positive Bacteria*							Penicillin G	Streptomycin
*SA*	18.2	19.4	17.5	16.9	13.2	24.7	29.4	25.1
*BS*	20.3	21.4	19.8	18.2	14.3	28.2	32.5	29.1
*Gram negative Bacteria*							Penicillin G	Streptomycin
*PA*	16.4	11.4	14.9	12.8	NA	22.8	28.3	24.3
*EC*	19.5	16.8	18.9	11.9	9.4	24.4	33.5	25.6

**Table 3:** Anti-microbial activities.

Microorganism	3d	3e	3f	3g	3h	ST. (30 µg/mL)	
*Fungi*						Itraconazole	Clotrimazole
*AF*	10.8	23.1	23.9	12.3	18.4	28.5	26.1
*GC*	10.2	22.3	17.2	11.4	19.6	27.1	23.1
*CA*	8.9	17.0	14.5	10.2	16.2	26.1	18.3
*SR*	NA	19.2	16.2	8.0	14.2	22.3	20.5
*Gram Positive Bacteria*						Penicillin G	Streptomycin
*SA*	10.5	23.2	18.5	12.9	14.4	29.4	25.1
*BS*	12.7	27.3	15.8	13.2	18.4	32.5	29.1
*Gram negative Bacteria*						Penicillin G	Streptomycin
*PA*	NA	21.2	19.9	11.0	NA	28.3	24.3
*EC*	8.5	24.8	20.9	10.8	13.8	33.5	25.6

**Table 4:** Anti-microbial activities.

Microorganism	4a	4b	4c	4d	4e	4f	ST. (30 µg/mL)	
*Fungi*							Itraconazole	Clotrimazole
*AF*	17.3	14.8	24.3	18.5	16.1	15.3	28.5	26.1
*GC*	18.4	13.9	22.4	19.1	17.1	16.2	27.1	23.1
*CA*	16.2	12.8	17.8	16.1	11.3	14.7	26.1	18.3
*SR*	17.9	NA	18.9	12.3	10.5	NA	22.3	20.5
*Gram Positive Bacteria*							Penicillin G	Streptomycin
*SA*	17.9	15.7	22.7	16.9	12.4	20.04	29.4	25.1
*BS*	18.2	17.2	25.2	19.7	13.5	22.08	32.5	29.1
*Gram negative Bacteria*							Penicillin G	Streptomycin
*PA*	12.3	NA	21.8	15.4	NA	12.7	28.3	24.3
*EC*	15.8	12.4	22.4	17.8	8.6	18.6	33.5	25.6

**Table 5:** Anti-microbial activities.

Microorganism	4g	4h	ST. (30 µg/mL)	
*Fungi*			Itraconazole	Clotrimazole
*AF*	14.8	20.2	26.1	28.5
*GC*	13.9	19.7	23.1	27.1
*CA*	12.4	16.8	18.3	26.1
*SR*	NA	11.3	20.5	22.3
*Gram Positive Bacteria*			Penicillin G	Streptomycin
*SA*	15.1	21.4	25.1	29.4
*BS*	16.4	26.1	29.1	32.5
*Gram negative Bacteria*			Penicillin G	Streptomycin
*PA*	NA	19.2	24.3	28.3
*EC*	10.4	20.9	25.6	33.5

## 3. Experimental

### 3.1. General

Melting points were determined uisng an Electrothermal Gallenkamp apparatus and are reported uncorrected. IR spectra were recorded in KBr using PyUnicam SP-1000 Spectrometer. ^1^H-NMR spectra were recorded using CDCl_3_ and DMSO-*d_6_* solutions using a Varian Em-300 MHz Spectrometer and chemical shifts are reported in ppm relative to that of TMS, which was used as an internal standard. Mass spectra were recorded using a AEI MS 30 mass spectrometer operating at 70 eV. Elemental analyses were carried out by the Microanalytical Center of Cairo University, Giza, Egypt. Antimicrobial activities were carried out at the Regional Center for Mycology and Biotechnology at Al-Azhar University, Cairo, Egypt.

### 3.2. Synthesis of Compounds **2a–h** and **3a–h**

Compounds **2a–h** and **3a–h**were prepared using previously described methods [22]. Compounds **2c** and **3c** are newly prepared and their physical constants, together with spectral and elemental analysis are shown below:

#### 3.2.1. *5-Acetyl-3-(4-bromophenyl)-2-imino-1,3,4-thiadiazole* (**2c**)

Gray solid, (80% yield), mp 160 °C (EtOH); IR (KBr) 3238 (NH), 1680 (C=O) cm^−1^; ^1^H-NMR (DMSO-*d_6_*) 2.46 (s, 3H, CH_3_), 7.37 (d, *J* = 9 Hz, 2H, ArH), 7.51 (d, *J* = 9 Hz, 2H, ArH), 10.75 (s, 1H, NH); MS *m/z* (%) 298 (M^+^, 2), 278 (13), 276 (42), 198 (25), 171 (25), 90 (54), 76 (4). Anal. Calcd. for C_10_H_8_BrN_3_OS (298.16): C, 40.28; H, 2.70; N, 14.09. Found: C, 40.08; H, 2.51; N, 14.00%.

#### 3.2.2. *N-[5-Acetyl-3-(4-bromophenyl)-3H-[1,3,4]-thiadiazol-2-ylidene]-benzamide* (**3c**)

Orange solid, (80% yield), mp 100 °C (EtOH); IR (KBr) 1686, 1608 (2C=O) cm^−1^; ^1^H-NMR (DMSO-*d_6_*) 2.66 (s, 3H, COCH_3_), 7.48–7.63 (m, 5H, ArH), 7.75 (d, *J* = 9 Hz, 2H, ArH), 8.01 (d, *J* = 9 Hz, 2H, ArH); MS *m/z* (%) 402 (M^+^, 1), 122 (50), 111 (13), 103 (23), 94 (35), 82 (41), 76 (100). Anal. Calcd. for C_17_H_12_BrN_3_O_2_S (402.27): C, 50.76; H, 3.01; N, 10.45. Found: C, 50.53; H, 3.11; N, 10.36%.

### 3.3. Synthesis of N-[3-aryl-5-(3-dimethylaminoacryloyl)-3H-[1,3,4]-thiadiazol-2-ylidene]-benzamides **4a–h**

A mixture of the appropriate 1,3,4-thiadiazole derivative **3** (10 mmol) and dimethylformamide-dimethylacetal (DMF-DMA) (2.4 g, 20 mmol) in dry toluene was stirred under reflux for 2 h. After cooling, methanol was added and the resulting solid was collected by filtration, washed with methanol, dried and crystallized from the appropriate solvent to afford the respective enaminones **4a–h**.

#### 3.3.1. *N-[3-(4-Methoxyphenyl)-5-(3-dimethylaminoacryloyl)-3H-[1,3,4]-thiadiazol-2-ylidene]-benzamide* (**4a**)

Orange solid, (90% yield), mp 260 °C (EtOH); IR (KBr), 1642, 1616 (2C=O) cm^−1^; ^1^H-NMR (DMSO-*d_6_*) 2.94 (s, 3H, CH_3_), 3.21 (s, 3H, CH_3_), 3.82 (s, 3H, OCH_3_), 5.81 (d, *J* = 12 Hz, 1H, =CH), 7.17 (d, *J* = 9 Hz, 2H, ArH), 7.45–7.59 (m, 5H, ArH), 7.83 (d, *J* = 9 Hz, 2H, ArH), 7.91 (d, *J* = 12 Hz, 9H, =CH). Anal. Calcd. for C_21_H_20_N_4_O_3_S (408.47): C, 61.75; H, 4.94; N, 13.72. Found: C, 61.53; H, 4.79; N, 13.65%.

#### 3.3.2. *N-[3-(4-Methylphenyl)-5-(3-dimethylaminoacryloyl)-3H-[1,3,4]-thiadiazol-2-ylidene]-benzamide* (**4b**)

Yellow crystals, (90% yield), mp 250 °C (EtOH/dioxane); IR (KBr) 1644, 1625 (2C=O) cm^−1^; ^1^H-NMR (DMSO-*d_6_*) 2.43 (s, 3H, CH_3_), 2.94 (s, 3H, CH_3_), 3,21(s, 3H, CH_3_), 5.82 (d, *J* = 12 Hz, 1H, =CH), 7.43–7.59 (m, 5H, ArH), 7.82 (d, *J* = 9 Hz, 2H, ArH), 7.91 (d, *J* = 12 Hz, 1H, CH=), 8.08 (d, *J* = 9 Hz, 2H, ArH). MS *m/z* (%) 392 (M^+^, 12), 104 (24), 85 (14), 77 (17), 98 (100), 97 (16), 104 (24), Anal. Calcd. for C_21_H_20_N_4_O_2_S (392.48): C, 64.27; H, 5.14; N, 14.27. Found: C, 64.43; H, 5.07; N, 14.38%.

#### 3.3.3. *N-[3-(4-Bromophenyl)-5-(3-dimethylaminoacryloyl)-3H-[1,3,4]-thiadiazol-2-ylidene]-benzamide* (**4c**)

Yellow solid, (80% yield), mp 125 °C (EtOH); IR (KBr) 1669, 1598 (2C=O) cm^−1^; ^1^H-NMR (DMSO-*d_6_*) 2.89 (s, 3H, CH_3_), 3.09 (s, 3H, CH_3_), 6.20 (d, *J* = 12 Hz, 1H, CH=), 7.30–7.41 (m, 9H, ArH), 7.60 (d, *J* = 12 Hz, 1H, CH=); MS *m/z* (%) 459 (M^+^, 1), 252 (100), 186 (70), 176 (54), 155 (59), 138 (60), 128 (36), 233 (84), 201 (61), 77(10). Anal. Calcd. for C_20_H_17_BrN_4_O_2_S (457.34): C, 52.52; H, 3.75; N, 12.25. Found: C, 52.30; H, 3.54; N, 12.09%.

#### 3.3.4. *N-[3-(4-Chlorophenyl)-5-(3-dimethylaminoacryloyl)-3H-[1,3,4]-thiadiazol-2-ylidene]-benzamide* (**4d**)

Orange solid, (80% yield), mp 320 °C (EtOH/dioxane); IR (KBr) 1643, 1619 (2C=O) cm^−1^; ^1^H-NMR (DMSO-*d_6_*) 2.68 (s, 3H, CH_3_), 2.97 (s, 3H, CH_3_), 5.82 (d, *J* = 12 Hz, 1H, =CH), 7.48–7.60 (m, 5H, ArH), 7.72 (d, *J* = 8 Hz, 2H, ArH), 7.93 (d, *J* = 12 Hz, 1H, =CH), 8.04 (d, *J* = 8 Hz, 2H, ArH); MS *m/z* (%) 415 (M^+^+2, 3), 413 (M^+^, 7), 111 (4), 98 (100), 77 (23). Anal. Calcd. for C_20_H_17_ClN_4_O_2_S (412.89): C, 58.18; H, 4.15; N, 13.57. Found: C, 58.02; H, 4.08; N, 13.46%.

#### 3.3.5. *N-[3-(4-Nitrophenyl)-5-(3-dimethylaminoacryloyl)-3H-[1,3,4]-thiadiazol-2-ylidene]-benzamide* (**4e**)

Yellow solid, (86% yield), mp 280 °C (EtOH/dioxane); IR (KBr) 1720, 1638 (2C=O) cm^−1^; ^1^H-NMR (DMSO-*d_6_*) 2.98 (s, 3H, CH_3_), 3.23 (s, 3H, CH_3_), 5.87 (d, *J* = 12 Hz, 1H, =CH), 7.49–7.63 (m, 5H, Ar-H), 7.93 (d, *J* = 12 Hz, 1H, =CH), 8.39 (d, *J* = 8 Hz, 2H, ArH), 8.51 (d, *J* = 8 Hz, 2H, ArH); MS *m/z* (%) 423 (M^+^, 5), 105 (40), 98 (100), 97 (11), 77 (31). Anal. Calcd. for C_20_H_17_N_5_O_4_S (423.45): C, 56.73; H, 4.05; N, 16.54. Found: C, 56.92; H, 4.08; N, 16.49%.

#### 3.3.6. *N-[3-Phenyl-5-(3-dimethylamino-acryloyl)-3H-[1,3,4]-thiadiazol-2-ylidene]-benzamide* (**4f**)

Orange Solid , (80% yield), mp 268 °C (EtOH); IR (KBr), 1641, 1624 (2C=O) cm^−1^; ^1^H-NMR (DMSO-*d_6_*): 5.83 (d, *J* = 12 Hz, 1H, =CH), 2.95 (s, 3H, CH_3_), 3.22 (s, 3H, CH_3_), 8.08 (d, *J* = 12 Hz, 1H, =CH) 7.46–7.98 (m, 10H, Ar-H); MS *m/z* (%): 378 (M^+^, 22), 361 (12), 331 (26), 105 (51), 98 (100), 77 (38). Anal. Calcd. for C_20_H_18_N_4_O_2_S (378.45): C, 63.47; H, 4.79; N, 14.80. Found: C, 63.50; H, 4.59; N, 14.67 %.

#### 3.3.7. *N-[3-(3-Methylphenyl)-5-(3-dimethylamino-acryloyl)-3H-[1,3,4]-thiadiazol-2-ylidene]-benzamide* (**4g**)

Orange solid, (82% yield), mp 200 °C (EtOH/Dioxane); IR (KBr) 1690, 1638 (2C=O) cm^−1^; MS *m/z* (%) 392 (M^+^, 11), 337 (24), 132 (35), 131 (22), 105 (100), 77 (69). Anal. Calcd. for C_21_H_20_N_4_O_2_S (392.48): C, 64.27; H, 5.14; N, 14.27. Found: C, 64.15; H, 5.01; N, 14.10%.

#### 3.3.8. *N-[3-(3-Chlorophenyl)-5-(3-dimethylamino-acryloyl)-3H-[1,3,4]-thiadiazol-2-ylidene]-benzamide* (**4h**)

Orange solid, (90% yield), mp 250 °C (EtOH); IR (KBr) 1646, 1639 (2C=O) cm^−1^; ^1^H-NMR (DMSO-*d_6_*) 2.97 (s, 3H, CH_3_), 3.23 (s, 3H, CH_3_), 5.81 (d, *J* = 12 Hz, 1H, =CH), 7.48–7.72 (m, 9H, ArH), 7.79 (d, *J* = 12 Hz, 1H, =CH); MS *m/z* (%) 415 (M^+^+2, 17), 413 (M^+^, 33), 395 (25), 329 (14), 98 (100), 105 (61), 77 (59). Anal. Calcd. for C_20_H_17_ClN_4_O_2_S (412.89): C, 58.18; H, 4.15; N, 13.57. Found: C, 58.20; H, 4.28; N, 13.64%.

### 3.4. Synthesis of N-[3-phenyl-5-(1H-pyrazol-3-yl)-3H-[1,3,4]thiadizol-2-ylidene]-benzamide (**5**)

A mixture of enaminone **4f** (1.89 g, 5 mmol) and hydrazine hydrate (5 mL) in absolute ethanol was stirred at reflux for 10 h and cooled. The formed solid was separated by filtration and crystallized from ethanol to give **5** as white solid, (65%, yield), mp 220 °C; IR (KBr) 3303 (NH), 1607 (C=O) cm^−1^; ^1^H-NMR (DMSO-*d_6_*) 6.82–7.51 (m, 10H, Ar-H), 7.58 (d, *J* = 8 Hz, 1H, pyrazole-H), 8.02 (d, *J*= 8 Hz, 1H, pyrazole-H), 9.21 (s, 1H, NH); MS *m*/*z* (%), 347 (M^+^,1), 257 (5), 235 (68), 132 (19), 103 (80), 90 (24), 76 (100). Anal. Calcd. for C_18_H_13_N_5_OS (347.40): C, 62.23; H, 3.77; N, 20.16. Found: C, 62.09; H, 3.54; N, 20.07%.

### 3.5. Synthesis of N-(5-isoxazol-5-yl-3-phenyl-3H-[1,3,4]-thiadiazol-2-ylidene)-benzamide (**6**)

To a solution of **4f** (1.89 g, 5 mmol) in absolute ethanol (20 mL) was added hydroxylamine hydrochloride (0.35 g, 5 mmol) and anhydrous potassium carbonate (0.5 g, 5 mmol). The mixture was stirred at reflux for 5 h, cooled and the precipitate formed was separated by filtration and crystallized from ethanol to give **6** as yellow solid, (70%, yield), mp 160 °C; IR (KBr) 1606 (C=O) cm^−1^; ^1^H-NMR (DMSO-*d_6_*) 7.36–7.72 (m, 10H, Ar-H), 7.95 (d, *J* = 8 Hz, 1H, isoxazole-H), 8.13 (d, *J* = 8 Hz, 1H, isoxazole-H); MS (*m*/*z*) ((% 348 (M^+^, 2), 121 (12), 105 (100), 91 (24), 77 (76). Anal. Calcd. for C_18_H_12_N_4_O_2_S (348.39): C, 62.06; H, 3.47; N, 16.08. Found: C, 61.98; H, 3.55; N, 16.11%.

### 3.6. Reaction of Enaminone **4f** with Active Methylene Compounds

#### 3.6.1. General Procedure

To a solution of **4f** (1.89 g, 5 mmol) in glacial acetic acid (20 mL) was added the corresponding active methylene compound (acetylacetone, ethyl acetoacetate, ethyl benzoylacetate or dibenzoyl methane) (5 mmol) and ammonium acetate (0.5 g, 6 mmol). The reaction mixture was stirred at reflux for 5–10 h (reaction progress monitored by using TLC) and poured into cold water. The formed solid was separated by filtration and crystallized from ethanol to give the respective products **8a–d**.

#### 3.6.2. *N-[5-(5-Acetyl-6-methyl-pyridin-2-yl)-3-phenyl-3H-[1,3,4]thiadiazol-2-ylidene]-benzamide* (**8a**)

Yellow solid, (80%, yield), mp 290 °C; IR (KBr), 1679, 1605 (2C=O) cm^−1^; ^1^H-NMR (DMSO-*d_6_*) 2.49 (s, 3H, CH_3_), 2.63 (s, 3H, COCH_3_), 7.47–7.72 (m, 10H, Ar-H), 8.04 (d, *J* = 8 Hz, 1H, ArH), 8.39 (d, *J* = 8 Hz, 1H, ArH); MS *m/z* (%) 415 (M^+^+1, 2), 414 (M^+^, 5), 136 (6), 104 (77), 90 (68), 76 (100). Anal. Calcd. for C_23_H_18_N_4_O_2_S (414.49): C, 66.65; H, 4.38; N, 13.52. Found: C, 66.49; H, 4.26; N, 13.28%.

#### 3.6.3. *6-(5-Benzoylimino-4-phenyl-4,5-dihydro-[1,3,4]-thiadiazol-2-yl)-2-methyl-nicotinic Acid Ethyl Ester* (**8b**)

Yellow solid, (80%, yield), mp 200 °C; IR (KBr) 1715, 1617 (2C=O) cm^−1^; ^1^H-NMR (DMSO-*d_6_*) 1.36 (t, *J* = 7 Hz, 3H, CH_3_), 2.83 (s, 3H, CH_3_), 4.35 (q, *J* = 7 Hz, 2H, CH_2_), 7.50–7.71 (m, 10H, Ar-H), 8.03 (d, *J* = 8 Hz, 1H, ArH), 8.35 (d, *J* = 8 Hz, 1H, ArH). MS *m/z* (%) 444 (M^+^, 5), 104 (82), 91 (51), 77 (100). Anal. Calcd.for C_24_H_20_N_4_O_3_S (444.52): C, 64.85; H, 4.54; N, 12.60. Found: C, 64.90; H, 4.35; N, 12.49 %.

#### 3.6.4. *6-(5-Benzoylimino-4-phenyl-4,5-dihydro-[1,3,4]-thiadiazol-2-yl)-2-phenyl-nicotinic Acid Ethyl Ester* (**8c**)

Orange solid, (80% , yield), mp 210 °C; IR (KBr) 1719, 1611 (2C=O) cm^−1^; ^1^H-NMR (DMSO-*d_6_*) 1.06 (t, *J* =7 Hz, 3H, CH_3_), 4.18 (q, *J*= 7 Hz, 2H, CH_2_), 7.46–8.11 (m, 15H, Ar-H), 8.22 (d, *J* = 7 Hz, 1H, ArH), 8.32 (d, *J* = 7 Hz, 1H, ArH); MS *m*/*z* (%) 506 (M^+^, 10), 117 (12), 105 (87), 77 (100). Anal. Calcd. for C_29_H_22_N_4_O_3_S (506.59): C, 68.76; H, 4.38; N, 11.06. Found: C, 68.53; H, 4.22; N, 11.20%.

#### 3.6.5. *N-[5-(5-Benzoyl-6-phenyl-pyridin-2-yl)-3-phenyl-3H-[1,3,4]thiadiazol-2-ylidene]-benzamide* (**8d**)

Yellow solid, (75%, yield), mp 175 °C; IR (KBr) 1669, 1618 (2C=O) cm^−1^; ^1^H-NMR (DMSO-*d_6_*) 7.49–7.94 (m, 20H, Ar-H), 8.02 (d, *J* = 8 Hz, 1H, pyridine-H), 8.20 (d, *J* = 8 Hz, 1H, pyridine-H); MS *m*/*z* (%) 539 (M^+^+1, 7), 538 (M^+^, 10), 222 (15), 105 (100), 98 (82), 91 (27), 77 (76). Anal. Calcd. for C_33_H_22_N_4_O_2_S (538.63): C, 73.59; H, 4.12; N, 10.40. Found: C, 73.44; H, 4.22; N, 10.23%.

### 3.7. Reaction of Enaminone **4f** with Heterocyclic Amines

#### 3.7.1. General Procedure

To a solution of **4f** (1.89 g, 5 mmol) in acetic acid (20 mL) was added the appropriate heterocyclic amine (5 mmol). The mixture was stirred at reflux for 6 h then cooled. The formed solid was separated by filtration and crystallized from the appropriate solvent.

#### 3.7.2. *5-(2-Benzoylimino-3-phenyl-1,3,4-thiadiazol-5-yl)-triazolo[1,5-a]pyrimidine* (**11**)

Yellow solid, (80%, yield), mp 300 °C (EtOH); IR (KBr) 1608 (C=O) cm^−1^; ^1^H-NMR (DMSO-*d_6_*) 7.45–8.10 (m, 10H, Ar-H), 8.11 (d, *J* = 4.5 Hz, 1H, ArH), 8.97 (s, 1H, triazole-H), 9.04 (d, *J* = 4.5 Hz, 1H, ArH); MS *m*/*z* (%) 399 (M^+^, 16), 229 (2.9), 173 (0.3), 105 (80), 77 (100); Anal. Calcd. for C_20_H_13_N_7_OS (399.44): C, 60.14; H, 3.28; N, 24.55. Found: C, 60.08; H, 3.21; N, 24.64%.

#### 3.7.3. *4-(2-Benzoylimino-3-phenyl-1,3,4-thiadiazol-5-yl)-benzimidazo[1,2-a]pyrimidine* (**12**)

Yellow solid, (80%, yield), mp 230 °C (Dioxane); IR (KBr) 1635 (C=O) cm^−1^; ^1^H-NMR (DMSO-*d_6_*) 7.46–7.69 (m, 14H, Ar-H), 7.93 (d, *J* = 4.5 Hz, 1H, pyrimidine-H), 8.08 (d, *J* = 4.5 Hz, 1H, pyrimidine-H); MS *m*/*z* (%), 448 (M^+^, 1), 377 (7), 105 (22), 98 (100), 77 (19). Anal. Calcd .for C_25_H_16_N_6_OS (448.51): C, 66.95; H, 3.60; N, 18.74. Found: C, 66.68; H, 3.51; N, 18.59%.

#### 3.7.4. *4-(2-Benzoylimino-3-phenyl-1,3,4-thiadiazol-5-yl)-7-phenyl-pyrazolo[1,5-a]pyrimidine* (**13**)

Yellow solid, (80%, yield), mp 280 °C (Dioxane); IR (KBr) 1606 (C=O) cm^−1^; ^1^H-NMR (DMSO-*d_6_*) 7.43–7.79 and 8.06–8.17 (m, 15H, Ar-H), 7.79 (d, *J* = 5 Hz, 1H, pyrimidine-H), 8.0 (s, 1H, pyrazole-H), 8.63 (d, *J* = 5 Hz, 1H, pyrimidine-H); MS *m*/*z* (%), 474 (M^+^, 39), 397 (16), 104 (100), 91 (30), 77 (77); Anal. Calcd. For C_27_H_18_N_6_OS (474.55): C, 68.34; H, 3.82; N, 17.71. Found: C, 68.20; H, 3.64; N, 17.56%.

### 3.8. Agar Diffusion Well Method to Determine the Antimicrobial Activity

The microorganism inoculums were uniformly spread using sterile cotton swab on a sterile Petri dish containing malt extract agar (for fungi) and nutrient agar (for bacteria). Each sample (100 μL) was added to each well (6 mm diameter holes cut in the agar gel, 20 mm apart from one another). The systems were incubated for 24–48 h at 37 °C (for bacteria) and at 28 °C (for fungi). After incubation, microorganism growth was observed. Inhibition of the bacterial and fungal growth were measured in mm. Tests were performed in triplicate [23].

## 4. Conclusions

In the investigation described above, a new series of 1,3,4-thiadiazole derivatives was prepared. In addition, 1,3,4-thiadiazole derivatives substituted at position-5 with heterocyclic rings were synthesized via reaction of enaminone **4f** with C- and N-nucleophiles. The antimicrobial properties of some of the prepared compounds were evaluated. The results demonstrate that selected members of this series, including **2b**, **3c** and **4c**, show excellent activities against all tested microorganisms compared with the standard fungicides itraconazole and clotrimazole and bactericides penicillin G and streptomycin.
